# Depressive Symptoms Associated with Decreased Choline Intake in Lactating Mothers of Preterm Infants

**DOI:** 10.3390/nu18111728

**Published:** 2026-05-28

**Authors:** Katherine Marie Ottolini, Gracie Spurney, Katherine Leah Wisner, Renee Geyao Pen, Julius Ngwa, Catherine Limperopoulos, Nickie Andescavage

**Affiliations:** 1 Developing Brain Institute, Children’s National Hospital, Washington, DC 20010, USA; kottolin@childrensnational.org (K.M.O.); graciespurney@gmail.com (G.S.); kwisner@childrensnational.org (K.L.W.); rpen@childrensnational.org (R.G.P.); jngwa@childrensnational.org (J.N.); climpero@childrensnational.org (C.L.); 2 Division of Neonatology, Children’s National Hospital, Washington, DC 20010, USA; 3 Department of Pediatrics, George Washington University School of Medicine, Washington, DC 20037, USA; 4 Department of Radiology, George Washington University School of Medicine, Washington, DC 20037, USA

**Keywords:** choline, prematurity, depression

## Abstract

**Background/Objectives**: Adequate choline intake is critical across the peripartum period for optimal maternal-offspring health. Maternal depressive symptoms are associated with poor dietary quality, but the relationship between postpartum depression and choline intake in lactating mothers of preterm infants, a period of heightened intake requirements, has not been previously explored. **Methods:** Lactating mothers of hospitalized preterm infants (born ≤36 weeks gestational age) completed depression screening with the Edinburgh Postnatal Depression Scale (EPDS) and a dietary choline intake survey. Mann–Whitney U tests compared maternal choline intake based on EPDS screen status (low-risk or at-risk for depression). Multivariable linear regression assessed the relationship between maternal depressive symptoms and choline intake. **Results**: EPDS screens were at-risk in 18 (42%) of the 43 participants. Median choline intake across the cohort was <50% of recommended dietary intake (median [IQR] 221 [147, 308] mg), with lower intake in mothers with at-risk EPDS screens compared to low-risk screens (median [IQR] score: 156 [105, 218] mg vs. 298 [196, 357] mg, *p* < 0.01). In multivariable linear regression, EPDS score was negatively associated with maternal choline intake (β [95% CI] = −0.13 [−0.26, −0.01], *p* = 0.03). **Conclusions**: Dietary choline intake is low among lactating mothers of preterm infants, particularly those with postpartum depressive symptoms.

## 1. Introduction

Choline is an essential micronutrient involved in widespread physiological processes [1,2,3,4,5]. Choline intake may have important implications for adult mental health and is inversely association with depressive symptoms [6]. This relationship is particularly pertinent for mothers of preterm infants who experience disproportionately high rates of postpartum depression [7,8,9,10,11,12,13,14,15]. Moreover, maternal choline demands are acutely increased during the latter half of pregnancy and postpartum during lactation, when choline transferred via the placenta and human milk plays a critical role in fetal and neonatal brain development including neurogenesis, synaptogenesis, and myelination [16,17,18]. Notwithstanding the importance of choline intake for maternal-infant health, the majority of pregnant and lactating women consume significantly less than the recommended daily intake of 450 mg/day during pregnancy and 550 mg/day during lactation [4,6,19,20,21].

Inadequate maternal choline intake during pregnancy places the developing fetus and infant at risk for deficiency, which may be further compounded by maternal choline undernourishment throughout lactation [22,23]. Preterm infants are particularly vulnerable to choline deficits as they are deprived of crucial third trimester placental micronutrient transfer [24,25]. Their reliance upon human milk as the primary nutritional source to meet these demands underscores the need for adequate maternal choline intake during lactation.

Despite the significance of maternal choline consumption during the perinatal period for both maternal mental health and infant outcomes, dietary intake in mothers of preterm infants remains poorly characterized, particularly in the context of postpartum depression [3,26,27,28]. We therefore examined the association between maternal depressive symptoms and choline intake in a cohort of lactating mothers of hospitalized preterm infants.

## 2. Materials and Methods

### 2.1. Participants

Lactating mothers of preterm infants (born ≤36 weeks gestational age) admitted to the Level IV neonatal intensive care unit (NICU) at Children’s National Hospital (Washington, DC, USA) between 2021 and 2026 were consecutively enrolled as part of a prospective, observational study. A preterm gestational age cutoff of ≤36 weeks was used to capture a cohort requiring more than a brief hospital stay and more prolonged management of prematurity-related comorbidities. As a freestanding children’s hospital, all infants were transferred from outlying birthing hospitals. Mothers of preterm infants admitted within the first month of life were eligible for enrollment if they were providing maternal milk to their infants (with or without donor human milk or preterm formula supplementation). Mothers of infants born with an underlying genetic syndrome, metabolic disorder, or perinatal central nervous system infection were excluded. Maternal race, ethnicity and education level were self-reported while the remainder of maternal and infant characteristics were extracted from the medical record including maternal age, parity, and psychiatric diagnoses and infant birth gestational age and weight, length of hospital stay, duration of invasive mechanical ventilation, and incidence of necrotizing enterocolitis or intraventricular hemorrhage (≥grade 2). This study was approved by the Children’s National Hospital Institutional Review Board, and informed written consent was obtained from all participants.

### 2.2. Questionnaires

Each participant was asked to complete self-reported postnatal depression screening and dietary choline intake survey during their infant’s hospitalization. Depression screening was performed using the Edinburgh Postnatal Depression Scale (EPDS), a validated 10-item questionnaire based on maternal symptoms within the preceding 7 days with scores ranging 0–30 [29]. For this high-risk population, an EPDS score threshold of ≥10 was used as an at-risk screen to increase sensitivity for detecting potential postpartum depression [12,29,30,31]. Respondents with an EPDS score of 10 greater were provided with additional mental health support including risk assessment, mental health referrals and/or establishment of a safety plan, as indicated. Average daily dietary choline intake during the preceding month was assessed using a semi-quantitative food frequency questionnaire based on 12 choline-rich foods (not including choline-containing supplement intake). This questionnaire was adapted from previously validated abbreviated choline surveys for use in a typical Western/U.S. diet [32,33,34,35]. Average daily intake was estimated using the approximate choline content (mg per serving) and reported consumption frequency (daily, weekly, or monthly/rarely) [32,33]. Adequacy of maternal daily choline consumption was divided into quartiles based on percentage of daily recommended intake consumed (550 mg/day for lactating mothers); Quartile 1 (Q1): <25% (<138 mg), Quartile 2 (Q2): 25–50% (138–275 mg), Quartile 3 (Q3): 50–75% (276–413 mg), and Quartile 4 (Q4): >75% (>413 mg) [4,21].

### 2.3. Statistical Analysis

Statistical analysis was performed using IBM SPSS Statistics version 28.0.0.0 [36]. Data normality was assessed using the Shapiro–Wilk Test. Participant characteristics were reported using medians with interquartile ranges (IQRs) for continuous variables and frequencies (percentages) for categorical variables. Univariate comparisons using Mann–Whitney U tests for continuous and Chi-square/Fisher’s exact tests for categorical measures were conducted to identify differences between maternal and infant characteristics and to compare choline intake between participants with low-risk and at-risk EPDS screens.

The relationship between maternal EPDS scores and the outcome of average choline intake (mg) was further assessed using multivariable linear regression. Maternal and infant characteristics that differed significantly by maternal EPDS screen status (low-risk vs. at-risk) or were independently associated with choline intake were included as covariates in the multivariable model, using an inclusion threshold of *p* < 0.10 given the modest sample size, with assessment for multicollinearity. All *p*-values were two-tailed, with a threshold *p* < 0.05 considered statistically significant and confidence intervals set at a 95% level.

## 3. Results

### 3.1. Characteristics by EPDS Screening Status

Of 113 enrolled participants in the overarching study, 86 (76%) mothers were lactating; of those, 43 (50%) completed concurrent choline intake surveys and EPDS screens during their infants’ hospitalizations. There were no significant differences in maternal age, education, or infant birth gestational age or weight between mothers who did and did not complete questionnaires. Median (IQR) maternal age of respondents was 32 (30, 34) years, with infants born at a median (IQR) gestational age of 28.7 (25.6, 32.0) weeks (Table 1). Infants of mothers with at-risk EPDS screens experienced a longer duration of hospital stay compared to those with low-risk screens (median [IQR] 86 [47, 158] days vs. 53 [32, 99] days, *p* = 0.09) and higher incidence of ≥grade 2 intraventricular hemorrhage (33% vs. 12%, *p* = 0.09). There were no significant differences in baseline maternal or infant clinical characteristics between mothers with and without at-risk EPDS screens (Table 1).

### 3.2. EPDS Score and Choline Intake

**Figure 1:**
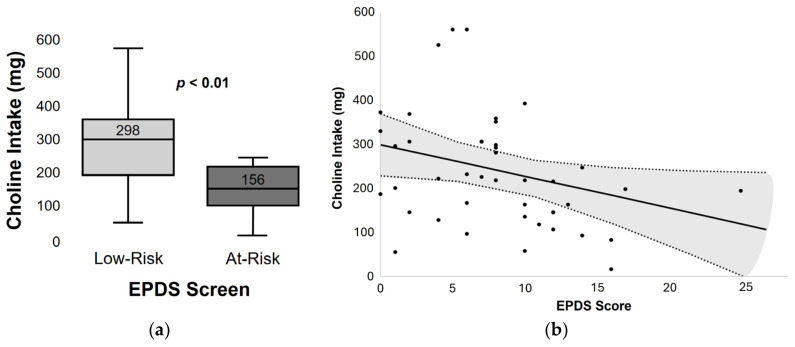
Relationship between maternal depressive symptoms and choline intake. (**a**) Median (IQR) choline intake based on maternal EPDS screen status; (**b**) maternal choline intake relative to EPDS score (unadjusted values).

Median (IQR) postnatal age at time of EPDS and choline intake survey completion was 46 (23, 73) days, with later postnatal age in mothers with at-risk compared to low-risk EPDS scores (median [IQR] 48 [26, 85] days vs. 30 [16, 59] days, *p* = 0.12) (Table 2). As expected, average EPDS scores were higher in the at-risk (18 mothers or 42%) relative to the low-risk mothers (median [IQR] EPDS score: 12 [10, 15] vs. 8 [2, 8], *p* < 0.01). Median choline intake was lower in those with at-risk EPDS scores compared to those with low-risk (median [IQR]: 156 [105, 218] mg vs. 298 [196, 357] mg, *p* < 0.01) (Figure 1a). Only 3 mothers (7% of the cohort) achieved choline intake within Q4, all with low-risk EPDS screens.

Choline intake was non-normally distributed and underwent necessary square root transformation prior to linear regression analysis (Figure 1b). In addition to maternal EPDS scores, the multivariable linear regression model included each infant’s postnatal age at survey completion, length of hospital stay, and presence of intraventricular hemorrhage (≥grade 2), with a model R^2^ of 0.27. Maternal EPDS score (β [95% CI] = −0.13 [−0.26, −0.01], *p* = 0.03) and infant postnatal age at survey completion (β [95% CI] = −0.03 [−0.06, −0.00], *p* = 0.03) were inversely related to choline intake.

## 4. Discussion

Lactating mothers of hospitalized preterm infants reported low overall intake of the essential micronutrient choline in this observational cohort. They also endorsed a high burden of postpartum depressive symptoms. Through this exploratory investigation, we identified a significant inverse relationship between maternal depressive symptoms and dietary choline intake. Whereas choline intake was inadequate in nearly all participants, lactating mothers with depressive symptoms consumed markedly less choline than those without, highlighting this group as particularly vulnerable for dietary choline deficiency.

Preterm delivery is an independent risk factor for maternal postpartum depression, with nearly two-fold greater odds of developing postpartum depression following preterm compared to healthy full-term birth [8]. In addition to increased rates of postpartum depression, mothers of preterm infants also experience more prolonged depressive symptoms [9,10]. The 42% incidence of elevated postpartum depression screens in our cohort was at the higher end of previously reported values [11,37]. This elevated rate could potentially reflect greater maternal psychological distress in response to higher infant illness severity associated with admission to a quaternary care NICU. This consideration may be supported by higher rates of intraventricular hemorrhage and longer hospital stays in infants of mothers with at-risk compared to low-risk EPDS screens within our cohort. Postnatal age at survey completion was also inversely associated with maternal choline intake within this cohort, highlighting the potential interrelatedness of these additional factors with maternal depressive symptoms and dietary choline consumption.

While postpartum depression has been linked to overall poor maternal diet quality in lactating women, this investigation specifically identified a negative association between depressive symptoms and choline intake during lactation [10,15]. The human body has a limited ability to produce endogenous choline, necessitating the exogenous consumption of choline-rich foods [3]. Lactation results in elevated choline requirements for the nutritional support of both mother and infant [3,4,21]. Consistent with a prior population-based study in Canadian lactating women, only 7% of mothers in our preterm cohort reported choline consumption within 75% of the daily requirement, all of whom had low-risk depression screens [19]. Dichotomization of this cohort based on maternal depression screen status revealed significantly lower choline intake in mothers with elevated depressive symptoms, of which 94% consumed less than half of the daily recommended intake compared to 40% of those with low-risk depression screens. Such inadequate choline intake could have important implications for the health of lactating mothers, as choline plays an integral role in adult liver, muscle, and neurobehavioral function [38,39,40,41,42]. Both animal and human studies implicate choline deficiency in the pathophysiology of depression, as reflected by altered cerebral choline concentrations within the hippocampus and prefrontal cortex [43,44,45,46,47].

Low choline intake in lactating mothers also poses potential risks for the development and long-term health of preterm infants. Preterm infants are born with inadequate choline stores, a problem compounded by the precipitous drop in plasma choline levels that occurs within days of birth [48,49]. Without the availability of placental nutrients, they are dependent upon the choline supplied by maternal milk to fuel critical choline-dependent neurodevelopmental processes [16,17,18,25,48]. Additionally, the choline content of multicomponent nutrient fortifiers routinely added to preterm maternal milk is not sufficient to achieve physiological plasma concentrations comparable to that of the developing fetus [50,51]. Evidence from interventional and observational trials indicates that increased maternal choline consumption correlates with higher concentrations in human milk [52,53,54]. Serum choline levels in preterm infants are closely related to consumption from maternal milk, highlighting the pivotal role of maternal dietary intake in supporting the health of their infants [22,25]. Greater prenatal maternal choline intake is linked to improved offspring attention and visuospatial memory through early childhood [27,55,56]. Such benefits from increased dietary choline intake in lactating mothers could potentially translate to significant improvements in long-term outcomes in preterm infants, who are at increased risk for neurodevelopmental impairment [57,58,59,60]. It is important to note these studies reflect more objective measures of choline status, which may be influenced by genetic variants in choline-metabolizing enzymes as well as the intake of vitamins involved in interrelated metabolic pathways, including B12 and folate [61,62,63]. The role of these additional, yet unmeasured factors, in this work should be considered when interpreting the strength of the association between maternal choline intake and depression scores within our cohort.

### Limitations

While this study is strengthened by the novelty of our research questions, several limitations are worth noting. Self-reported survey responses can lead to both recall and selection bias. We assessed depressive symptoms via the EPDS, which is a screening tool and nondiagnostic for depression. Additionally, use of a self-reported, abbreviated food frequency questionnaire may have underestimated total choline intake with a similar risk of recall bias. Moreover, without biomarker corroboration, this work could not account for variation in maternal serum choline levels related to individual metabolism and intake of related micronutrients, underscoring the need for further study. Outside of the food frequency questionnaire, we did not collect data on specific maternal choline supplement intake, which may further underestimate maternal dietary choline. It also is possible for residual confounding from other covariates not assessed within our analysis, particularly regarding maternal socioeconomic status and overall diet quality. The small sample size also limited our statistical power and the number of potential covariates that could be considered within our statistical models. Our cohort was restricted to mothers of infants admitted to a single-center quaternary care NICU, potentially limiting the generalizability of these results. Survey results also reflected a cross-sectional observation, limiting causal inference. Collectively, these limitations highlight the need for larger, multicenter studies that integrate longitudinal measures of choline intake, biomarkers of choline status, and formal mental health diagnostics.

## 5. Conclusions

This exploratory study reveals a critical deficiency in dietary choline intake among lactating mothers of preterm infants, particularly those experiencing postpartum depressive symptoms. These findings suggest a link between maternal postpartum depressive symptoms and inadequate choline consumption, emphasizing the need for targeted nutritional support and heightened mental health vigilance. Further research is required to validate these preliminary findings within a larger population and develop interventions that simultaneously address mental health and nutritional adequacy in mothers of preterm infants. Validation through future multicenter studies in mothers of preterm infants, spanning the continuum of intrauterine and extrauterine choline exposure and incorporating serum measures of choline metabolism, may afford actionable clinical interventions to nutritional and mental health screenings.

## Figures and Tables

**Table 1:** Participant characteristics of total cohort and delineated by EPDS screening status.

Characteristic	Total Cohort (n = 43)	EPDS Screening Status		*p*-Value *
		Low-Risk Score < 10 (n = 25)	At-Risk Score 10–30 (n = 18)	
Maternal				
Age (years), median (IQR)	32 (30, 34)	32 (31, 34)	32 (28, 33)	0.29
Primiparity, n (%)	20 (47)	12 (48)	8 (44)	0.82
Race, n (%)				0.29
White	20 (47)	11 (44)	9 (50)	
Asian/Pacific Islander	1 (2)	0 (0)	1 (6)	
Black/African American	15 (35)	8 (32)	7 (39)	
Multiple/Other	7 (16)	6 (24)	1 (6)	
Hispanic/Latino Ethnicity, n (%)	7 (16)	5 (20)	2 (11)	0.39
Education				0.80
Less Than High School	3 (7)	2 (8)	1 (6)	
High School	11 (26)	6 (24)	5 (28)	
Any College	16 (37)	9 (36)	7 (39)	
Graduate Degree	11 (26)	8 (32)	3 (17)	
Not Reported	2 (5)	0 (0)	2 (11)	
Psychiatric Diagnosis, n (%)	10 (23)	7 (28)	3 (17)	0.39
Infant				
Birth Gestational Age (weeks), median (IQR)	28.7 (25.6, 32.0)	28.9 (25.9, 31.7)	28.2 (25.5, 32.3)	0.88
Birth Weight (kg), median (IQR)	1.05 (0.83, 1.56)	1.03 (0.86, 1.67)	1.08 (0.80, 1.61)	0.81
Length of Hospital Stay (days), median (IQR)	66 (33, 113)	53 (32, 99)	86 (47, 158)	0.09
Invasive Mechanical Ventilation (days), median (IQR)	6 (0, 38)	3 (0, 33)	8 (0, 59)	0.56
Necrotizing Enterocolitis, n (%)	10 (23)	5 (20)	5 (28)	0.55
Intraventricular Hemorrhage ≥ Grade 2, n (%)	9 (21)	3 (12)	6 (33)	0.09

* Mann–Whitney U or Chi-Square/Fisher’s Exact Test.

**Table 2:** Choline intake and EPDS scores of total cohort and delineated by EPDS screening status.

Characteristic	Total Cohort (n = 43)	EPDS Screening Status		*p*-Value *
		Low-Risk Score < 10 (n = 25)	At-Risk Score 10–30 (n = 18)	
Depression Screening				
Postnatal Age (days), median (IQR)	46 (23, 73)	30 (16, 59)	48 (26, 85)	0.12
EPDS Score				
Median (IQR)	8 (4, 12)	8 (2, 8)	12 (10, 15)	<0.01
Range	0–25	0–8	6–25	
Choline Intake				
Choline intake (mg)				
Median (IQR)	221 (147, 308)	298 (196, 357)	156 (105, 218)	<0.01
Range	18–564	56–564	18–396	
Recommended Dietary Choline Intake Quartile, n (%)				<0.01
Q1 (<25%): <138 mg	9 (21)	3 (12)	6 (33)	
Q2 (25–50%): 138–275 mg	18 (42)	7 (28)	11 (61)	
Q3 (50–75%): 276–413 mg	13 (30)	12 (48)	1 (6)	
Q4 (>75%): >413 mg	3 (7)	3 (12)	0 (0)	

* Mann Whitney U or Chi-Square/Fisher’s Exact Test.

## Data Availability

Deidentified individual participant data (including data dictionaries) will be made available, in addition to study protocols, the statistical analysis plan, and the informed consent form. The data will be made available upon publication to researchers who provide a methodologically sound proposal for use in achieving the goals of the approved proposal. Proposals should be submitted to nniforat@childrensnational.org.

## Abbreviations

The following abbreviations are used in this manuscript:

EPDS	Edinburgh Postnatal Depression Scale
IQR	Interquartile Range
NICU	Neonatal Intensive Care Unit
